# Farmed and Wild Macroalgae as a Safe Source of Macro and Trace Elements

**DOI:** 10.3390/biology15110820

**Published:** 2026-05-22

**Authors:** Tomás Chainho, Rui Cereja, Alícia Pereira, Vera Marques, João C. Silva, Sofia Pessanha, Pedro Reis Costa, António Marques

**Affiliations:** 1 IPMA, I.P.—Portuguese Institute for the Sea and Atmosphere, I. P., Avenida Doutor Alfredo Magalhães Ramalho, nº 6, 1495-165 Algés, Portugal; 2 MARE—Marine and Environmental Sciences Centre, Faculty of Sciences, University of Lisbon, Campo Grande, 1749-016 Lisbon, Portugal; 3 CIIMAR—Interdisciplinary Centre of Marine and Environmental Research, University of Porto, Terminal de Cruzeiros do Porto de Leixões, Av. General Norton de Matos s/n, 4450-208 Matosinhos, Portugal; 4 Laboratory for Instrumentation, Biomedical Engineering and Radiation Physics, LA-REAL.NOVA School of Science and Technology, Campus FCT-UNL, 2829-516 Caparica, Portugal; 5 Centre of Marine Sciences (CCMAR/CIMAR LA), University of Algarve, Campus de Gambelas, 8005-139 Faro, Portugal

**Keywords:** seaweeds, food-safety, seasonality, elemental profile, marine biotoxins

## Abstract

The increasing popularity of macroalgae products highlights their potential as a safe source of essential macro and microelements for consumers. This study characterized wild and farmed macroalgae elemental composition and assessed the presence of biotoxins to identify potential health risks. Wild and farmed macroalgae samples were collected throughout 2024–2025, from three aquaculture production sites along the Portuguese coast (Ria de Aveiro, Matosinhos, and Olhão). Samples were freeze-dried, compressed and using a micro-XRF spectrometer, elemental composition was assessed. Macroalgae species factor was the strongest driver of elemental composition, explaining over 80% variation in macro and trace elements. Origin did not showcase statistical significance for elemental composition. Seasonal differences, though relatively small in extent, significantly impacted redox-sensitive elements in macroalgae, namely copper and manganese. All elements stayed below 30% of the recommended dietary recommendations, except iron (ranging from 0.5% to 111.8% of UL%) and Mn (ranging from 0.1% to 101% of UL%). All samples revealed the absence of regulated marine toxins. Only traces of the non-regulated cyclic-imine toxin SPX1 were detected in samples of *Fucus vesiculosus*. All quantified elements do not represent any risk for human health, strengthening the safety of macroalgae from the Portuguese coast.

## Simple Summary

Seaweed products are rising within the food industry, but consumers need clear information about their nutritional value and safety. This study examined wild and farmed samples collected from three areas of the Portuguese coast to understand whether species, origin, or season affected their elemental composition and the presence of biotoxins. The results showed that the species factor had the strongest effect on element composition, while farmed and wild samples showed no major differences. All macro and trace elements analyzed were below dietary limits. No regulated marine toxins were detected in any sample. These findings highlight the potential that Portuguese seaweeds represent a safe food resource and support their use in sustainable aquaculture production.

## 1. Introduction

In the past decades, worldwide aquaculture production has risen, confirming the relevance of this sector as a key element of the “Blue Transformation” agenda [1]. In the context of aquaculture, macroalgae play a central role in promoting the development of the sector. Macroalgae production has increased due to the global need for sustainable, low-trophic food and novel products with functional compounds that can be used by pharmaceuticals, nutraceuticals, cosmetics, agriculture, food, and feed. Macroalgae also have a big relevance in coastal management, due to their capabilities to provide ecosystem services, preserve biodiversity and drive the economic development [2]. As sentinel species, macroalgae play an important role in monitoring environmental quality, due to their capacity to bioaccumulate and bioremediate chemical contaminants, including heavy metals [3,4]. Such capabilities make them a valuable tool for assessing the integrity of shorelines, particularly against anthropogenic threats, such as eutrophication and pollution [5,6,7]. In addition, they also have biofiltering capabilities, absorbing dissolved nitrogen and phosphorus, mitigating eutrophication and converting waste nutrients into usable biomass [8]. Furthermore, macroalgae products can be nutrient-rich and environmentally sustainable, providing bioactive and functional ingredients with wide applicability in l food and nutraceuticals [9,10]. Additionally, macroalgae’s broader role as eco-friendly biofertilizers and aquafeed ingredients has been expanding, as reflected in novel applications by the industry [11,12,13,14,15,16].

Although these applications demonstrate the importance of macroalgae in food supply chains and aquaculture, the accumulation of elements that go beyond regulated levels also has to be taken into account [17,18]. Studies involving macroalgae such as *Fucus* sp., *Ascophyllum* sp., and *Enteromorpha* sp. reveal substantial differences in their element accumulation behaviour (particularly As, Cd, Hg and Pb), due to different cell wall composition, surface area, and growth rate [3,5,19]. Additionally, bioaccumulation capabilities are influenced by seasonal changes related to environmental parameters such as temperature and nutrient availability [3,4]. Therefore, owing to their high level of pollutant bioaccumulation potential, safety concerns remain regarding the use of macroalgae biomass for agricultural or food purposes [3,20].

Among contaminants that macroalgae can bioaccumulate, arsenic and toxins from harmful algal blooms (HABs), such as domoic acid, saxitoxins, okadaic acid and other lipophilic toxins, can be of great concern for consumers [21,22,23]. The dynamics of toxins from HABs are largely driven by seasonality, with the occurrence of peaks in concentration levels due to environmental factors, such as temperature, nutrient availability, and hydrodynamics. Understanding this variability of contaminants is a vital framework towards minimizing risks related to public health hazards and for deploying effective monitoring programmes in coastal management [22].

Considering the increasing incorporation of macroalgae in food, pharmaceutical, and cosmetic products, there is an urgent need for comprehensive assessment of their nutritional relevance and safety. By evaluating macro and trace elements and assessing potential risks for consumers, this research fills a significant knowledge gap within the Portuguese coast context and provides essential insights to the industry that can guide risk assessment frameworks, inform regulatory policies, and support the sustainable development of macroalgae based products.

The main objective of this study is to assess in different macroalgae, how species, origin (wild vs. farmed) and seasonal variability influence the levels of macro and trace elements alongside the presence of lipophilic toxins from HABs, such as okadaic acid and dinophysistoxins, azaspiracids, yessotoxins, and cyclic imines, as well as ASP (amnesic shellfish poisoning) and PSP (paralytic shellfish poisoning) toxins, using the Portuguese coast as case study, while also evaluating the potential implications for consumers.

## 2. Materials and Methods

### 2.1. Sampling

**Figure 1:**
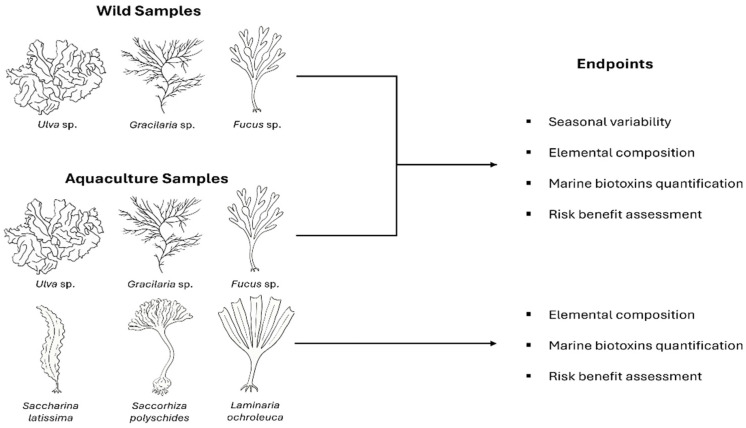
Experimental design and corresponding endpoints.

Six macroalgae species were collected in Ria de Aveiro Lagoon (wild) and from aquaculture facilities (Ria de Aveiro, Matosinhos, Olhão): (1) wild and farmed *Ulva* sp., *Fucus* sp. and *Gracilaria* sp. were collected in Ria de Aveiro; and (2) *Laminaria ochroleuca*, *Saccorhiza polyschides*, and *Saccharina latissima* were sampled from an aquaculture site in Matosinhos and Olhão (Figure 1). They were analyzed to address four key endpoints: (1) seasonal variability (for *Ulva* sp., *Gracilaria* sp. and *Fucus* sp.), (2) elemental composition, (3) marine biotoxin quantification and (4) risk-benefit assessment. Seasonal samplings were performed for wild and farmed macroalgae in Ria de Aveiro, with three replicates per sample.

Each biological replicate consisted of a pooled sample of three individual macroalgae collected at the same site and throughout the seasons 2024–2025, minimizing intra-group variability and better representing the seaweed population. Moisture content was determined from an adapted methodology of Guiné and Barroca [24]. Samples were weighed immediately after collecting (fresh weight, FW) using an EMS 300-3 (Kern, Balingen, Germany) precision scale and subsequently frozen at −80 °C. The samples were then freeze-dried in a ScanVac CoolSafe freeze dryer (Labogene, Lillerød, Denmark) for 24 h at 0.06 mbar and −52 °C, after which they were immediately transferred to a desiccator and stored until a second weighing was performed (dry weight, DW). The dried algal biomass was subsequently homogenized to a fine, homogeneous powder using a knife mill (Grindomix GM 200, Retsch, Haan, Germany) prior to further analysis.

### 2.2. Elemental Quantification

Freeze-dried macroalgae samples were compressed into small tablets (0.57 ± 0.07 g each) using a 10-ton hydraulic press (Specac, Orpington, UK). A benchtop micro-XRF spectrometer, the M4 TORNADO by Bruker (Berlin, Germany), was then used. The system makes use of a low-power X-ray tube with a Rh anode, operated at 50 kV and 500 µA, and a poly-capillary lens focused the beam on a 40 µm spot for Mo-Ka emission lines. Two operating modes were undertaken: using a 12.5 µm Al filter between the X-ray tube and the samples to improve the signal-to-noise ratio of the lower region of the spectrum, and using a combination of filters 100 µm Al/50 µm Ti/25 µm Cu to improve the signal-to-noise ratio in the middle region of the spectrum [25]. The analyses were performed using an area of interest of 10 × 10 mm^2^, and point-by-point measurements were performed on samples with a step size of 40 µm and 10 ms acquisition time per pixel, followed by the retrieval of the cumulative spectrum. Limits of Detection and Limits of Quantification were calculated according to Ensina et al. [26] for DORM-4 and TORT-3 Certified Reference Materials (CRM) (Table S2).

Quantitative analysis was performed using the in-built software (MQuant) based on the Fundamental Parameters method [27] and applying the methodology described by Manguinhas et al. [28] using a matrix of 9% H, 30% C and 40% O. The validation of the method for DORM-4 and TORT-3 CRMs is shown in Supplementary Table S1. Elements analyzed using this method were: K, Ca, Mn, Fe, Cu, Zn, As, Br and Sr.

### 2.3. Toxin Analysis

#### 2.3.1. Toxin Extraction

All freeze-dried samples (1.0 ± 0.1 g) from all origins were placed into 50 mL polypropylene centrifuge tubes, and 20 mL of 100% methanol was added for lipophilic toxins, 20 mL of 50% methanol was added for ASP toxins analysis, and 20 mL of 1% acetic acid was added for PSP toxins analysis, and mixed thoroughly on a vortex for 5 min. Following this procedure, the tubes were centrifuged at ≥ 3600 *g* for 10 min, and the supernatant was filtered through a 0.2 μm syringe filter and stored at −20 °C until liquid chromatography analysis.

#### 2.3.2. Lipophilic Toxins Determination by Liquid Chromatography with Tandem Mass Spectrometry Detection (LC-MSMS)

The LC-MSMS equipment consisted of an Agilent 1290 Infinity liquid chromatograph coupled to a triple quadrupole mass spectrometer Agilent 6470 (Agilent Technologies, Waldbronn, Germany). The chromatographic separation was conducted as described in Braga et al. [29] with a Zorbax SB- C8 RRHT column (2.1 × 50 mm, 1.8 μm), protected with a guard column (2.1 × 5 mm, 1.8 μm). The mobile phase A was water with 2 mM ammonium formate (Sigma-Aldrich, St. Louis, MO, USA) and 50 mM formic acid (LC-MS, Carlo Erba, Emmendingen, Germany); the water was purified using a Milli-Q 185 Plus system from Millipore (Oeiras, Portugal). Mobile phase B was 95% acetonitrile (LC-MS grade, Carlo Erba, Germany) with 2 mM ammonium formate and 50 mM formic acid. An elution gradient at a flow rate of 0.4 mL/min was used as follows: 0–3 min, gradient from 88 to 50% eluent A; 3–6.5 min, gradient from 50 to 10% eluent A; 6.5–8.9 min, 10% eluent A; 8.9–10 min, gradient from 10 to 88% eluent A. The detection was carried out in Multiple Reaction Monitoring (MRM) acquisition mode. Two MRM transitions were monitored for each compound from the following toxin groups: OAs—okadaic acid, AZAs—azaspiracid, YTX—yessotoxin, SPXs—spirolides, GYMs—gymnodimines, and PnTXs—pinnatoxins (see Supplementary Materials, Table S1). Five calibration standard solutions (0.5–12 ng/mL) were set up for quantification using a matrix match calibration and certified reference standards purchased from CIFGA (Lugo, Spain).

#### 2.3.3. Amnesic Shellfish Poisoning (ASP) Toxins Determination by Liquid Chromatography with UV Detection (HPLC-UV)

HPLC analysis was performed as described in Costa et al. [30] on a Hewlett-Packard (HP) Model 1290 Infinity equipped with a quaternary pump, autosampler, oven and diode-array detector (DAD). The column used was a Nucleosil 100-5 C18 (125 × 3 mm, 5 µm), with a guard-column Lichrospher 100 RP-18 (4 × 4 mm, 5 µm). Detection wavelength was set at 242 nm with a 10 nm bandwidth, and reference wavelength at 450 nm with a 100 nm bandwidth. A confirmatory wavelength of 262 nm was used. Calibration was conducted using a certified DA standard (0.5–10 µg/mL), purchased from CIFGA (Lugo, Spain). 

#### 2.3.4. Paralytic Shellfish Poisoning (PSP) Toxins Determination by Liquid Chromatography with Fluorescence Detection (HPLC-FLD)

HPLC-FLD was based on the precolumn oxidation method developed by Lawrence and Niedzwiadek [31]. The HPLC-FLD equipment consisted of a Hewlett-Packard/Agilent Model 1290 Infinity quaternary pump, autosampler (Agilent, Santa Clara, CA, USA), column oven, and Model 1260 Infinity fluorescence detector (Agilent, Santa Clara, CA, USA). The paralytic shellfish toxin oxidation products were separated using a reversed-phase Supelcosil LC-18, 15 × 4.6, 5 μm column (Supelco, Sigma-Aldrich, Sintra, Portugal). The mobile phase gradient consisted of 0–5% B (0.1 M ammonium formate in 5% acetonitrile, pH 6) in the first 5 min, 5–70% B for the next 4 min, and back to 0% B in the next 2 min. Then, 100% mobile phase A (0.1 M ammonium formate, pH 6) was used for 3 min before the next injection. Flow rate was 1 mL/min, and the detection wavelength was set to 340 nm for excitation and 395 nm for emission, according to Costa et al. [32]. Calibration was carried out using certified PSP toxins standards, purchased from CIFGA (Lugo, Spain).

### 2.4. Nutritional Values

To evaluate the nutritional implications of macro and trace elements, mean dry-weight (DW) concentrations (mg kg^−1^ DW ± SD) of farmed and wild samples from Ria de Aveiro, as well as farmed macroalgae from Olhão and Matosinhos, were converted to a fresh-weight (FW) using species-specific conversion factors calculated using the following formula:

F = 100/(% Dry Weight)

The average dry weight content of macroalgae samples was 13.90% for *Fucus* sp., 19.43% for *Gracilaria* sp., 15.20% for *Ulva* sp., 14.1% for *L. ochroleuca*, 12.91% for *S. polyschides*, and 13.93% for *S. latissima*, corresponding to conversion factors of 7.19, 5.15, 6.58, 7.09, 7.75 and 7.18, respectively. Estimated FW concentrations (mg kg^−1^ FW) were then compared against adult Tolerable Upper Intake Levels (UL) (maximum chronic intake from all sources unlikely to produce adverse effects) established by EFSA [33], to determine if a consumption of 50 g FW of macroalgae would exceed the safe daily intakes of six regulated elements (calcium (Ca), copper (Cu), iron (Fe), manganese (Mn), potassium (K) and zinc (Zn)). Adult UL and children UL (4–10 years) were used.

To complement the risk assessment, estimated intakes were also compared with EFSA dietary reference values, including Population Reference Intakes (PRI) and Adequate Intakes (AI), for both adults and children (4–10 years). These values represent intake levels that meet the nutritional requirements of most healthy individuals. For iron (Fe) and copper (Cu), sex specific reference values were applied for adults, while for zinc (Zn), different dietary phytate levels were considered to account for variations in bioavailability.

Inorganic arsenic (As) was also assessed against the maximum permissible level for seaweed-based foods of 3.0 mg kg^−1^ DW [34].

### 2.5. Statistical Analysis

All statistical analyses were performed in R 4.4.3 (R Core Team, Vienna, Austria) [35]. To characterize temporal dynamics, samples were grouped by Season (spring, summer, autumn, winter). For each combination of Origin × Species × Element, seasonal effects were assessed using one-way ANOVA at α = 0.05. Prior to ANOVA, homogeneity of variances was assessed using Levene’s test (car 3.1-2). Whenever significant effects were detected, Tukey’s Honest Significant Difference (HSD) post hoc comparisons were applied using the agricolae 1.3-8 package. To evaluate interspecific differences in elemental composition across the six macroalgae species (*Fucus* sp., *Gracilaria* sp., *Laminaria ochroleuca*, *Saccharina latissima*, *Saccorhiza polyschides*, and *Ulva* sp.), a series of one-way ANOVAs was conducted for each element, treating Species as a fixed factor. Post hoc tests were performed as described above, and all values were reported as mean ± standard deviation (SD). To quantify the relative contribution of species, season, and origin to the overall elemental variance within wild and farmed samples from Ria de Aveiro, a variance partitioning analysis was conducted using linear mixed-effects models (lmer, lme4 package). Each model was fit separately for each element using the formula:

Value ~ 1 + (1 | Origin) + (1 | Origin: Species) + (1 | Season)

Models were fitted using restricted maximum likelihood (REML). The percentage of variance explained by each factor (Origin, Species (within Origin), Season, and Residual) was calculated by dividing the variance of each component by the total variance and multiplying by 100. This approach allowed the identification of dominant sources of variability for each element, supporting ecological and physiological interpretation. All data wrangling and reshaping were performed using dplyr (v1.1.4), tidyr (v1.3.0), and reshape2 (v1.4.4). Data visualization was carried out with ggplot2 (v3.5.0).

## 3. Results

### 3.1. Macro and Trace Elements

#### 3.1.1. Variance Across Species, Season, and Origin

**Figure 2:**
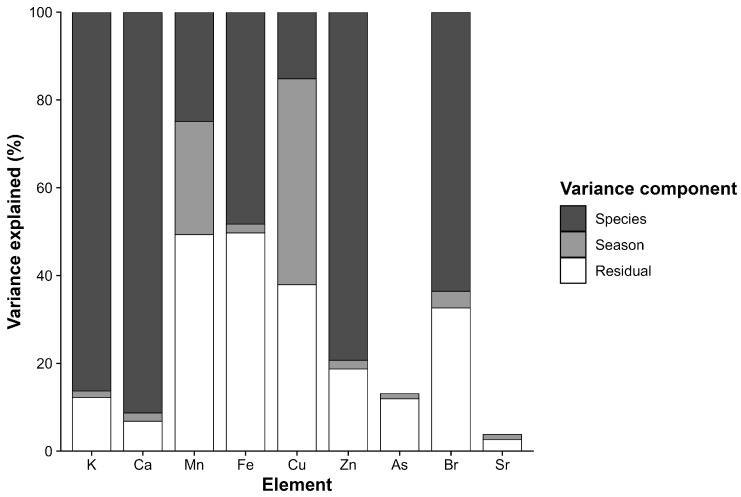
Percentage (%) of variance in wild and farmed samples from Ria de Aveiro (*Ulva* sp., *Gracilaria* sp. and *Fucus vesiculosus*) calculated as the proportion of each variance component relative to the total variance, extracted from a linear mixed-effects model using restricted maximum likelihood (REML). Potassium (K), calcium (Ca), manganese (Mn), iron (Fe), copper (Cu), zinc (Zn), arsenic (As), bromine (Br), and strontium (Sr).

Regarding the variance for the elements among biological and environmental factors (Figure 2), species was the major factor for most of the elements. Strontium (Sr) had the highest proportion of variance explained by species (96.2%), followed by calcium (Ca) (91.2%), arsenic (As) (87.0%), and potassium (K) (86.3%), with negligible effects of season and origin. Zinc (Zn) and bromine (Br) also had significant proportions of variance explained by species (79.3 and 63.5%, respectively), along with significant proportions of residuals. Manganese (Mn) and copper (Cu) had more evenly distributed proportions. For manganese, species explained 24.9% of the variance, while season explained 25.8%. Residuals explained the remaining proportion of the variance (49.3%). For copper, the major proportion of the variance was explained by season (46.9%), with species and residuals explaining smaller proportions (15.2 and 37.9%, respectively). Iron (Fe) had similar proportions of variance explained by species and residuals (48.3 and 49.7%, respectively). There were no significant proportions of the variance explained for the factor origin.

#### 3.1.2. Species

Significant interspecific differences in elemental composition were observed among macroalgae species (Table 1). When looking into K content, which ranged from 12,023 to 40,354 mg/kg Dw, *Gracilaria* sp. showed the highest concentrations, followed by intermediate levels in *L. ochroleuca*, whereas lower K concentrations were found in *Fucus* sp. and *S. latissima*, and the lowest values were recorded in *Ulva* sp. (*p* < 0.05). Highest Ca content levels, which ranged from 1023 to 4932 mg/kg Dw, were present in *L. ochroleuca*, whereas *Gracilaria* sp. had notably lower Ca concentrations (*p* < 0.05). Manganese (Mn) accumulation, which ranged from 1.35 to 287 mg/kg Dw, was significantly higher in *Fucus* sp., followed by *Gracilaria* sp., and the remaining species exhibited minimal Mn content.

Iron (Fe) content varied significantly, ranging from 29.7 to 1458 mg/kg Dw, with *Gracilaria* sp. showing the highest concentrations, intermediate levels occurred in *Fucus* sp., whereas *L. ochroleuca* and *S. latissima* had significantly lower Fe concentrations (*p* < 0.05). Copper (Cu), which ranged from 2.13 to 3.73 mg/kg Dw, had no statistically significant differences among species (*p* > 0.05). Zinc (Zn) concentrations differed notably, ranging from 4.91 to 56.6 mg/kg Dw, with significantly higher levels observed in *Fucus* sp., while the remaining species showed significantly lower concentrations (*p* < 0.05). Highest As concentrations, which ranged from 1.46 to 29.1 mg/kg Dw, occurred in *Fucus* sp., followed by *S. latissima* and *L. ochroleuca*, whereas the lowest concentrations were recorded in *Ulva* sp. (*p* < 0.05). Bromine (Br) concentrations, which ranged from 172 to 425 mg/kg Dw, were highest in *S. latissima* and *Fucus* sp., and significantly lower in *Ulva* sp. (*p* < 0.05). Finally, strontium (Sr), which ranged from 15.0 to 434 mg/kg Dw, exhibited significantly higher concentrations in *Fucus* sp., intermediate values in *L. ochroleuca* and *S. latissima*, and lower concentrations were recorded for *Ulva* sp. and *Gracilaria* sp. (*p* < 0.05).

#### 3.1.3. Origin

Origin comparisons within species did not show statistical differences between Ria de Aveiro farmed and wild samples (Table 2 and Table S3). In *Fucus* sp., K ranged from 18,053 to 22,127 mg/kg Dw and was higher in farmed than wild samples (*p* < 0.05), while Fe ranged from 357 to 605 mg/kg Dw and was greater in wild than farmed *Fucus* sp. samples (*p* < 0.05). In *Gracilaria* sp., no significant origin effect was observed for K, Ca, Mn, or Fe (*p* > 0.05), but wild *Gracilaria* sp. exhibited higher Zn than farmed samples, ranging from 9.30 to 16.7 mg/kg Dw (*p* < 0.05). Farmed *Ulva* sp. had higher K, ranging from 10,012 to 14,035 mg/kg Dw, and lower Ca, ranging from 1927 to 2705 mg/kg Dw, than wild samples (*p* < 0.05). No other significant differences between origins were detected for Cu, As, Br, or Sr in any species (*p* > 0.05).

#### 3.1.4. Seasonality

##### Farmed Macroalgae

**Figure 3:**
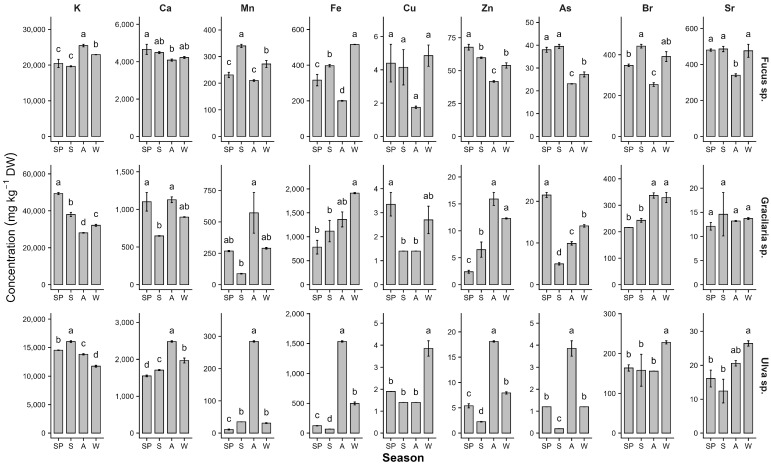
Seasonal variation in concentrations (mg kg^−1^ DW) in farmed *Fucus* sp., *Gracilaria* sp., and *Ulva* sp. from Ria de Aveiro. Abbreviations: potassium (K); calcium (Ca); manganese (Mn); iron (Fe); copper (Cu); zinc (Zn); arsenic (As); bromine (Br); strontium (Sr); spring (SP); summer (S); autumn (A); winter (W). Different lowercase letters in each column indicate statistically significant differences among seasonal-origin combinations for each species (Tukey HSD, *p* < 0.05).

*Gracilaria* sp. exhibited marked seasonal fluctuations in elemental composition (Figure 3). K, ranging from 28,065 to 49,351 mg/kg DW, was highest in spring, intermediate in summer and winter, and lowest in autumn (*p* < 0.05). Ca, ranging from 648 to 1128 mg/kg DW, was higher in autumn and spring, and lowest in summer (*p* < 0.05). Manganese, ranging from 87.5 to 572 mg/kg DW, peaked in autumn and was lowest in summer (*p* < 0.05). Fe, ranging from 784 to 1912 mg/kg DW, was highest in winter and lowest in spring (*p* < 0.05). Cu, ranging from 1.40 to 3.35 mg/kg DW, peaked in spring and was lower in summer and autumn (*p* < 0.05). Zn, ranging from 2.40 to 15.9 mg/kg DW, was highest in autumn and winter, intermediate in summer and lowest in spring (*p* < 0.05). As, ranging from 5.00 to 21.5 mg/kg DW, was highest in spring and lowest in summer (*p* < 0.05). Br, ranging from 216 to 337 mg/kg DW, peaked in autumn and winter and was lowest in spring and summer (*p* < 0.05). In contrast, Sr, ranging from 12.1 to 14.6 mg/kg DW, showed no significant seasonal variations (*p* > 0.05).

In *Fucus* sp. (Figure 3), K, ranging from 19,642 to 25,465 mg/kg DW, was highest in autumn and lowest in spring and summer (*p* < 0.05). Ca, ranging from 4086 to 4662 mg/kg DW, peaked in spring and was lowest in autumn (*p* < 0.05). Mn, ranging from 210 to 340 mg/kg DW, peaked in summer and was lowest in autumn and spring (*p* < 0.05). Fe, ranging from 200 to 516 mg/kg DW, was highest in winter and lowest in autumn (*p* < 0.05). Zn, ranging from 41.8 to 67.8 mg/kg DW, peaked in spring and was lowest in autumn (*p* < 0.05). As, ranging from 23.1 to 39.5 mg/kg DW, was highest in spring and summer, and lowest in autumn (*p* < 0.05). Br, ranging from 254 to 442 mg/kg DW, peaked in summer and was lowest in autumn (*p* < 0.05). Sr, ranging from 340 to 486 mg/kg DW, was highest in summer, spring and winter (*p* < 0.05). In contrast, Cu, ranging from 1.75 to 4.85 mg/kg DW, showed no significant seasonal variations (*p* > 0.05).

*Ulva* sp. (Figure 3) had the highest K, ranging from 11,758 to 16,042 mg/kg DW, in summer and the lowest in winter (*p* < 0.05). Calcium, ranging from 1552 to 2481 mg/kg DW, peaked in autumn and was lowest in spring (*p* < 0.05). Mn, ranging from 10.9 to 284 mg/kg DW, peaked in autumn and was lowest in spring (*p* < 0.05). Fe, ranging from 66.5 to 1534 mg/kg DW, was highest in autumn and lowest in summer (*p* < 0.05). Cu, ranging from 1.40 to 3.85 mg/kg DW, peaked in winter and was lower in the remaining seasons (*p* < 0.05). Zn, ranging from 2.25 to 18.1 mg/kg DW, peaked in autumn and was lowest in summer (*p* < 0.05). As, ranging from 0.200 to 3.85 mg/kg DW, was highest in autumn and lowest in summer (*p* < 0.05). Br, ranging from 156 to 228 mg/kg DW, was elevated in winter and lowest in the remaining seasons (*p* < 0.05). At last, Sr, ranging from 12.4 to 26.4 mg/kg DW, peaked in winter and was lowest in summer and spring (*p* < 0.05).

##### Wild Macroalgae

**Figure 4:**
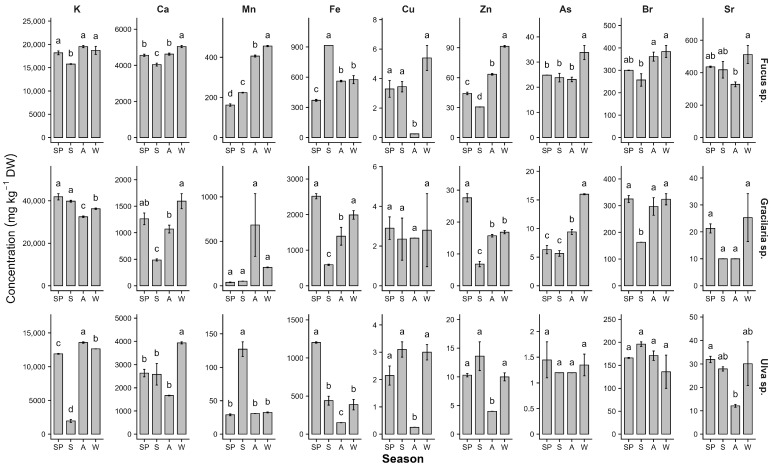
Seasonal variation in concentrations (mg kg^−1^ DW) in wild *Fucus* sp., *Gracilaria* sp., and *Ulva* sp. from Ria de Aveiro samples. Abbreviations: potassium (K); calcium (Ca); manganese (Mn); iron (Fe); copper (Cu); zinc (Zn); arsenic (As); bromine (Br); strontium (Sr); spring (SP); summer (S); autumn (A); winter (W). Different lowercase letters in each column indicate statistically significant differences among seasonal-origin combinations for each species (Tukey HSD, *p* < 0.05).

*Gracilaria* sp. showed pronounced seasonal trends (Figure 4). Potassium, ranging from 32,461 to 41,872 mg/kg DW, was highest in spring and summer, and lowest in autumn (*p* < 0.05). Calcium, ranging from 486 to 1596 mg/kg DW, peaked in winter and was lowest in summer (*p* < 0.05). Iron, ranging from 590 to 2516 mg/kg DW, was higher in spring and lowest in summer (*p* < 0.05). Zinc, ranging from 6.80 to 27.6 mg/kg DW, peaked in spring and was lowest in summer (*p* < 0.05). Arsenic, ranging from 5.65 to 16.0 mg/kg DW, was highest in winter and lowest in summer and spring (*p* < 0.05). Bromine, ranging from 163 to 325 mg/kg DW, was reduced in summer and highest in the remaining seasons (*p* < 0.05). In contrast, Mn, ranging from 38.5 to 684 mg/kg DW, Cu, ranging from 2.35 to 2.90 mg/kg DW, and Sr, ranging from 10.0 to 25.3 mg/kg DW, showed no significant seasonal variations (*p* > 0.05).

In *Fucus* sp. (Figure 4), K, ranging from 15,780 to 19,542 mg/kg DW, reached minimum levels in summer, and were higher in the remaining seasons (*p* < 0.05), Ca, ranging from 4041 to 5044 mg/kg DW, was highest in winter and lowest in summer (*p* < 0.05), Fe, ranging from 370 to 915 mg/kg DW, was highest in summer and lowest in spring (*p* < 0.05), Mn, ranging from 162 to 456 mg/kg DW, peaked in winter and was lowest in spring (*p* < 0.05), Cu, ranging from 0.250 to 5.40 mg/kg DW, revealed lower values in autumn, and were higher in the remaining seasons (*p* < 0.05), and Br, ranging from 257 to 384 mg/kg DW, peaked in winter and autumn, and was lower in summer (*p* < 0.05). Winter peaks were also observed for Zn, ranging from 30.7 to 91.4 mg/kg DW, and the lowest in summer (*p* < 0.05), As, ranging from 23.1 to 33.8 mg/kg DW, the lowest in the remaining seasons (*p* < 0.05), and Sr, ranging from 328 to 512 mg/kg DW, the lowest in autumn (*p* < 0.05).

*Ulva* sp. (Figure 4) reached the highest K peak, ranging from 1960 to 13,568 mg/kg DW, in autumn and the lowest in summer (*p* < 0.05), Ca, ranging from 1669 to 3935 mg/kg DW, was higher in winter and the lowest in the remaining seasons (*p* < 0.05), Fe, ranging from 151 to 1202 mg/kg DW, was higher in spring and the lowest in the remaining seasons (*p* < 0.05), Mn, ranging from 29.0 to 127 mg/kg DW, was higher in summer and the lowest in the remaining seasons (*p* < 0.05), Cu, ranging from 0.250 to 3.10 mg/kg DW, and Zn, ranging from 4.00 to 13.6 mg/kg DW, were higher in spring, summer and winter (*p* < 0.05), and Sr, ranging from 12.1 to 31.9 mg/kg DW, was higher in spring and the lowest in autumn (*p* < 0.05). In contrast, As, ranging from 1.20 to 1.45 mg/kg DW, and Br, ranging from 136 to 196 mg/kg DW, showed no significant seasonal variations (*p* > 0.05).

#### 3.1.5. Nutritional Contribution

Across all species, Ca, Cu and Zn tolerable Upper Intake Levels (UL) from a 50 g FW portion were consistently below 5% (Table 3) for adults. A similar trend was observed for children (4–10 years), with these elements contributing only marginally to UL, remaining well below levels of concern (Table 3).

In contrast, Mn and Fe showed higher UL contributions and interspecific differences. For *Fucus* sp., Mn comprised 32% and 38% of the tolerable upper limit (UL) in farmed and wild samples, respectively, while Fe comprised 9–15% of UL. Similarly, in *Gracilaria* sp., Mn comprised 23–29% of the UL, and Fe comprised 25–31%; wild samples were higher than farmed samples in both species. In *Ulva* sp., Mn comprised 5–8% and Fe comprised 9–10% of the UL. In *Laminaria ochroleuca*, *S. latissima*, and *S. polyschides*, low values were detected in all samples; none exceeded more than 1.5% of the UL.

For children aged 4–10 years, Mn comprised 61% and 46% of the UL in *Fucus* sp. and *Gracilaria* sp., respectively; Fe comprised 82% of the UL in *Gracilaria* sp., which comprised the highest values in this group of elements studied.

For dietary reference intakes (DRI), Mn comprised the highest values in all species studied and reached 85–101% of adequate intake in adults, while it surpassed 100% in children in both *Fucus* sp. and *Gracilaria* sp., especially in wild samples. Fe comprised high values, especially in *Gracilaria* sp., which surpassed 100% in both adult men and women and children. On the contrary, Ca, Cu, and Zn comprised less than 10% in all species studied for both adults and children.

### 3.2. Macroalgae Safety

#### 3.2.1. Biotoxins

In all samples, none of the EU-regulated lipophilic toxins (OAs, AZAs and YTXs), and ASP and PSP toxins were detected. However, in the brown macroalga *Fucus vesiculosus* the non-regulated cyclic-imine toxin 13-desmethyl spirolide C (SPX1) was identified. SPX1 was present in every sample, but only at very low (Autumn and Summer) or trace concentrations (spring and winter), regardless of the origin (Table 4).

#### 3.2.2. Arsenic

Total arsenic concentration varied within origin (Table 5), with the highest values detected in *Fucus* sp. (aquaculture samples (31.9 ± 7.4 mg/kg DW); wild samples (26.3 ± 4.8 mg/kg DW). Lower concentrations were present in *Gracilaria* sp. (9.34–12.6 mg/kg DW), while *Ulva* sp. showed lower levels (1.30–1.61 mg/kg DW). Assuming that 10% of total arsenic is present as inorganic arsenic (iAs) [36], estimated iAs concentrations ranged from 0.13 ± 0.02 to 3.19 ± 0.74 mg/kg DW. When comparing these results to regulated limits for inorganic arsenic (3.0 mg/kg DW set by CEVA [34]), *Fucus* sp. aquaculture samples exceeded the regulatory limit (106.3%), whereas wild samples remained below (87.7%). All *Gracilaria* sp. and *Ulva* sp. samples were below the limit, representing 31.1–42.0% and 4.3–5.4% of the regulatory threshold, respectively.

## 4. Discussion

### 4.1. Variations in Macro and Trace Elements

Species was the dominant factor influencing the elemental composition of macroalgae, explaining over 80% of the variance for several elements (i.e., K, Ca, Sr, As, Zn). Indeed, macroalgae accumulated the levels of As, Sr, Ca, and Br differently, according to species. Brown macroalgae, like *Fucus* sp., generally reveal higher accumulation levels of these elements, similar to results reported previously [7,20,37] likely due to their richness in alginates and fucoidans. Recent studies by Warnasooriya et al. [38] and Biancarosa et al. [39] also corroborate the importance of species-specific physiology in the accumulation of macro and trace elements, stressing the valorization of macroalgae as a pillar of the “Blue Transformation” agenda for global food security. In addition, the elevated metal accumulation capacity of some brown algae, like *Fucus* sp., highlights their potential as environmental bioindicators [3,17,38], reinforcing the strategic value of species selection for aquaculture and food applications.

Elements related to oxidative stress and metabolic activity, like Cu and Mn, demonstrated considerable fluctuation in accordance with seasonal changes [40,41], where species was a determining factor. This is in accordance with earlier studies [4,5,7] that highlighted the impact of environmental variability, such as nutrient availability and water temperature, on photosynthetic activity and trace metal uptake, particularly for redox-sensitive elements. Interestingly, wild samples displayed higher elemental fluctuation across seasons than those from aquaculture systems. This difference may reflect the selection of aquaculture sites in areas with lower environmental loads of macro and micro elements, resulting in reduced elemental accumulation, corroborating the FAO [1] reports on the stabilizing positive effect of aquaculture production regarding food safety. Furthermore, *Fucus* sp. and *Gracilaria* sp. wild samples presented generally lower Cu and Mn concentrations during Spring and/or Summer seasons, i.e., when higher temperatures and light conditions occur, being consistent with previous studies linking element seasonality with seasonal macroalgae growth [37,42].

Residual variability for Mn, Fe, Cu, and Br points to the role of environmental factors or biological interactions not accounted for in the current methodological approach. Similar complexities are highlighted by previous studies that point out similar complexities [3,4,37,43], emphasizing the role of biotic as well as abiotic factors in the composition of elements in macroalgae. Elements such as Mn, Cu, and Fe, which are often related to antioxidant enzyme systems, may be especially responsive to short-term stressors such as salinity or UV fluctuations [38]. In contrast, origin had no significant influence on elemental variability, marking both farmed and wild samples with similar quality for consumers. Nevertheless, samples were based on pooled material from three individuals, which improved group-level representativeness but prevented assessment of individual variability, which can have some interest to the industry.

### 4.2. Nutritional Contribution and Safety

Macroalgae produced under sustainable conditions contribute to a balanced diet due to their content of macro and trace elements, though these organisms can also accumulate contaminants. The macroalgae studied represent a relevant source of trace elements such as Mn and Fe, whereas other studies also reported high levels of Cu, Zn and Ca in seaweeds, which are often limited in plant-based or processed foods [44], supporting their classification as functional foods with potential to help address human and animal nutritional requirements worldwide [40,45,46]. Differentiation among species was also found for macro and trace elements composition, reinforcing distinct applicability, since *Gracilaria* sp. and *Fucus* sp. revealed higher levels of Fe and Mn, while *Ulva* sp. showed lower accumulation and wider safety margins. Concerning DRI, *Fucus* sp. and *Gracilaria* sp. can provide substantial contributions to essential mineral intake of Mn, whereas *Ulva* sp. showed slightly lower but consistent contributions, and *L. ochroleuca* and *S. polyschides* revealed limited contributions to the DRI of adults and children. Despite some elements (Mn and Fe) exceeding the dietary reference levels (DRI), none of the elements studied exceeded tolerable upper levels (UL). This finding suggests macroalgae can serve as a supplementary Mn source. It should be noted that the nutritional exposure estimates relied on a 50 g FW, not accounting for rehydration and boiling processes, which may alter real dietary exposure [47]. Mn plays an important role in bone formation and enzymatic catalysis. Although high levels of Mn could potentially cause neurotoxicity, the observed content is below the UL limits, indicating a beneficial yet controlled contribution to an individual’s daily dietary intake [48,49]. When compared to dietary reference intake (DRI) values, Mn showed the highest contribution among all elements and was close to or exceeded adequate levels for *Fucus* sp. and *Gracilaria* sp., highlighting their potential as valuable dietary sources of these essential minerals.

Concerning 4 to 10-year-old children, similar results were obtained, with significantly above adequate levels for all species for Mn. This suggests that macroalgae products have a great relative nutritional value for Mn without adverse effects under realistic consumption scenarios. This finding is in line with other studies, which also identify that the potential risk of adverse health effects from seaweed consumption could be specific to some species and elements, but not to all seaweeds [36,50,51]. It is important to consider that the elemental quantification by micro-XRF relied on certified reference materials and targeted a defined group of elements, so the risk-benefit assessment did not include other relevant seaweed contaminants or nutrients (e.g., iodine, cadmium, lead, mercury, and nickel). Further studies in these areas could have a broader scope of elements, so more conclusions regarding bioaccumulation and food safety could be made.

In this study, macroalgae also showed the capacity to accumulate toxic metals such as arsenic. Only *Fucus* sp. slightly exceeded the regulatory limit established by AFSSA [52] by approximately 6%, but not the other seaweeds studied. It is important to note that these estimates are based on mean values and an estimated ratio of inorganic arsenic, and therefore, variability among samples may influence compliance with the regulatory threshold. The study also identified negligible risk from intake of EU-regulated lipophilic toxins through seaweeds, including OAs, AZAs and YTXs. Only the nonregulated cyclic imine toxin 13 dimethyl spirolide C (SPX1) was detected at trace levels in *Fucus vesiculosus*, and not in the remaining samples. These levels do not indicate a risk to human health, despite the known neurotoxic mechanism of this compound through inhibition of cholinergic receptors [53].

## 5. Conclusions

This study shows that elemental composition in macroalgae depends strongly on species. *Fucus vesiculosus* showcased higher arsenic, manganese, calcium, strontium, and zinc levels than the other species examined, whereas *Gracilaria* sp. revealed higher levels of potassium, manganese and iron, *L. ochroleuca* and *S. latissima* had higher content of calcium, and *S. polyschides* showed higher levels of potassium and bromine. This pattern supports their use in different applications requiring distinct elemental profiles. Seasonal variation plays a smaller role, yet changes in copper and manganese point to seasonal shifts in metabolic activity and oxidative stress. Sampling across different seasons remains necessary to ensure consistent data for both composition and safety. A second pattern appears when comparing origins, with aquaculture samples showing narrower ranges than wild samples. This indicates that conditions with lower environmental loads of macro and trace elements reduce environmental variability and lead to a more stable elemental profile. Nutritional contribution becomes clearer when results are interpreted against dietary reference intakes. Indeed, *Fucus* sp. and *Gracilaria* sp. stand out due to their manganese and iron content, both at levels that reach or exceed adequate intake, but not reaching the UL for both origins. This places them in contrast with *Ulva* sp., *L. ochroleuca*, *S. latissima* and *S. polyschides*, which show lower contributions and accumulation.

Toxin data reveal that macroalgae are safe for consumers, with only the non-regulated SPX1 being detected in *Fucus* sp. samples, and always at trace levels. In contrast, arsenic follows a distinct pattern, with *Fucus* sp. being the sole species exceeding the regulatory limits of this element in seaweed when a precautionary approach is implemented (i.e., estimation of 10% inorganic arsenic and 50 g consumption). Hence, an increase in dietary intake could lead to a higher exposure to iAs. Overall, all seaweeds assessed, from both wild and farmed origins, can be incorporated into a healthy and balanced diet. Nevertheless, given their capacity to bioaccumulate, careful and consistent regulatory monitoring is needed. As the global market for macroalgae products expands, our study underscores the importance of comprehensive and responsive regulatory controls to ensure the safety of consumers’ health and promote sustainable aquaculture sector development.

## Figures and Tables

**Table 1:** Elemental concentrations (K, Ca, Mn, Fe, Cu, Zn, As, Br, and Sr) (mg/kg Dw; mean ± STDEV) across macroalgae species. Values within each element column, followed by distinct lowercase letters, indicate statistically significant differences based on Tukey’s test (*p* < 0.05). Potassium (K), calcium (Ca), manganese (Mn), iron (Fe), copper (Cu), zinc (Zn), arsenic (As), bromine (Br), and strontium (Sr).

Species	K	Ca	Mn	Fe	Cu	Zn	As	Br	Sr
*Fucus* sp.	20,090 ± 2897 ^c^	4465 ± 334 ^ab^	287 ± 100 ^a^	481 ± 210 ^bc^	3.44 ± 1.72 ^a^	56.6 ± 18.1 ^a^	29.1 ± 6.7 ^a^	342 ± 66 ^b^	434 ± 70 ^a^
*Gracilaria* sp.	37,238 ± 6440 ^a^	1023 ± 341 ^d^	274 ± 252 ^a^	1458 ± 635 ^a^	2.41 ± 0.90 ^ab^	13.0 ± 7.7 ^b^	10.9 ± 5.6 ^c^	279 ± 63 ^bc^	15.0 ± 5.9 ^c^
*Ulva* sp.	12,023 ± 4160 ^d^	2316 ± 769 ^c^	72.5 ± 89.2 ^b^	550 ± 518 ^b^	2.13 ± 1.13 ^ab^	8.94 ± 5.09 ^b^	1.46 ± 1.02 ^d^	172 ± 31 ^d^	22.2 ± 8.1 ^c^
*Laminaria ochroleuca*	25,553± 2952 ^b^	4932 ± 1748 ^a^	2.92 ± 2.14 ^b^	37.0 ± 16.4 ^c^	2.77 ± 0.61 ^ab^	4.91 ± 0.65 ^b^	17.4 ± 3.5 ^b^	231 ± 13 ^c^	236 ± 54 ^b^
*Saccharina latissima*	18,172 ± 282 ^cd^	4641 ± 368 ^ab^	1.35 ± 0.92 ^b^	51.5 ± 0.7 ^bc^	2.25 ± 0.49 ^ab^	7.85 ± 0.49 ^b^	22.8 ± 1.6 ^ab^	355 ± 12 ^ab^	207 ± 7 ^b^
*Saccorhiza polyschides*	40,354 ± 1750 ^a^	3322 ± 279 ^bc^	2.00 ± 0.00 ^b^	29.7 ± 2.6 ^c^	3.73 ± 1.15 ^a^	5.58 ± 2.47 ^b^	6.12 ± 1.80 ^cd^	425 ± 15 ^a^	246 ± 32 ^b^

**Table 2:** Origin comparisons between Ria de Aveiro Aquaculture (A) and wild (W) samples (K, Ca, Fe, and Zn) (mg/kg Dw; mean ± STDEV) (*Ulva* sp., *Gracilaria* sp. and *Fucus vesiculosus*). Values within each element column are followed by lowercase letters for each species origin, indicating statistically significant differences based on Tukey’s test (*p* < 0.05). Potassium (K), calcium (Ca), iron (Fe), and zinc (Zn).

Species	Origin	K	Ca	Fe	Zn
*Fucus* sp.	A	22,127 ± 2476 ^a^	4364 ± 262	357 ± 124 ^b^	55.8 ± 10.2
*Fucus* sp.	W	18,053 ± 1541 ^b^	4565 ± 383	605 ± 210 ^a^	57.4 ± 24.4
*Gracilaria* sp.	A	36,889 ± 8578	943 ± 211	1294 ± 454	9.30 ± 5.62 ^b^
*Gracilaria* sp.	W	37,586 ± 3877	1103 ± 437	1622 ± 773	16.7 ± 7.94 ^a^
*Ulva* sp.	A	14,035 ± 1650 ^a^	1927 ± 377 ^b^	555 ± 629	8.43 ± 6.35
*Ulva* sp.	W	10,012 ± 5011 ^b^	2705 ± 882 ^a^	545 ± 423	9.46 ± 3.81

**Table 3:** Percentage of the Tolerable Upper Intake Level (%UL) and Dietary Reference Intake (%DRI) for adults (Ad) and children (Ch) (4–10 years old) resulting from the consumption of 50 g fresh weight (FW) of macroalgae. Values are calculated from mean FW concentrations of calcium (Ca), manganese (Mn), iron (Fe), copper (Cu), and zinc (Zn), for farmed (A) and wild (W) specimens of *Fucus* sp., *Gracilaria* sp. and *Ulva* sp. and *L. ochroleuca*, *S. latissima*, and *S. polyschides*. Tolerable Upper Intake Levels (ULs, mg/day FW) were taken from EFSA [33] and represent the maximum chronic daily intake from all dietary sources that is unlikely to pose a risk of adverse health effects in adults. Dietary reference values (DRI or AI, mg/day) were taken from the European Food Safety Authority (EFSA). For iron (Fe) and copper (Cu), the sex-specific reference values for the worst-case scenario (lowest level) were applied, i.e., men for Fe and women for Cu. For zinc (Zn), values are presented for the worst-case scenario, i.e., with the lowest dietary phytate level, reflecting its influence on zinc bioavailability.

Element	UL/DRI FW	(mg/Day) Ad (Ch)	*Fucus* sp. A (%UL) Ad (Ch)	*Fucus* sp. W (%UL) Ad (Ch)	*Gracilaria* sp. A (%UL) Ad (Ch)	*Gracilaria* sp. W (%UL) Ad (Ch)	*Ulva* sp. A (%UL) Ad (Ch)	*Ulva* sp. W (%UL) Ad (Ch)	*L. ochroleuca* A (%UL) Ad (Ch)	*S. polyschides* A (%UL) Ad (Ch)	*S. latissima* A (%UL) Ad (Ch)
Ca	UL FW	2500 (1500)	1.7 (2.8)	1. 8 (3.0)	0.3 (0.5)	0.3 (0.6)	0.5 (0.9)	0.8 (1.3)	1.4 (2.3)	0.9 (1.4)	1.3 (2.3)
	DRI FW	950 (800)	4.5 (5.3)	4. 7 (5.6)	0.8 (0.9)	0.9 (1.1)	1.4 (1.7)	2.0 (2.3)	3.7 (4.4)	2.3 (2.7)	3.4 (4.0)
Mn	UL FW	8.0 (5.0)	32.0 (51.1)	38.0 (60.7)	28.9 (46.2)	23.3 (37.3)	7.8 (12.5)	4.8 (7.6)	0.3 (0.4)	0.2 (0.3)	0.1 (0.2)
	DRI FW	3.0 (1.5)	85.2 (170.4)	101.2 (202.4)	77.1 (154.1)	62.1 (124.3)	20.8 (41.6)	12.7 (25.3)	0.7 (1.4)	0.4 (0.9)	0.3 (0.6)
Fe	UL FW	40.0 (15.0)	8.8 (23.5)	14.8 (39.5)	24.5 (65.3)	30.8 (82.0)	9.7 (25.7)	9.5 (25.2)	0.7 (1.8)	0.5 (1.3)	0.9 (2.4)
	DRI FW	11.0 (11.0)	32.0 (32.0)	53.8 (53.8)	89.1 (89.1)	111.8 (111.8)	35.1 (35.1)	34.4 (34.4)	2.4 (2.1)	1.8 (1.8)	3.3 (3.3)
Cu	UL FW	5.0 (2.0)	0.7 (1.9)	0.6 (1.5)	0.3 (0.9)	0.4 (1.0)	0.3 (0.8)	0.3 (0.7)	0.4 (1.0)	0.5 (1.2)	0.3 (0.8)
	DRI FW	1.3 (1.0)	2.9 (3.7)	2.3 (3.0)	1.3 (1.7)	1.5 (2.0)	1.2 (1.5)	1.1 (1.5)	1.5 (2.0)	1.9 (2.4)	1.2 (1.6)
Zn	UL FW	25 (10.0)	2.2 (5.5)	2.2 (5.6)	0.3 (0.7)	0.5 (1.3)	0.2 (0.6)	0.3 (0.7)	0.1 (0.4)	0.2 (0.4)	0.2 (0.6)
	DRI FW	9.4 (7.4)	5.8 (7.4)	6.0 (7.6)	0.7 (1.0)	1.4 (1.7)	0.6 (0.8)	0.7 (0.9)	0.4 (0.5)	0.4 (0.5)	0.6 (0.7)

**Table 4:** Concentrations of the toxin SPX1 detected in the wild and farmed brown macroalga *Fucus* sp. harvested in Ria de Aveiro, expressed as ng g^−1^ dry weight. LQ, limit of quantification (10.7 ng L^−1^).

Season (Year)	Sample Origin	SPX1
Autumn (2024)	Aquaculture	1.5
	Wild	1.8
Spring (2024)	Aquaculture	<LQ
	Wild	<LQ
Summer (2024)	Aquaculture	0.9
	Wild	0.9
Winter (2025)	Aquaculture	<LQ
	Wild	<LQ

**Table 5:** Percentage of the regulatory limit (3.0 mg/kg DW (EFSA, [33]) level for inorganic arsenic (iAs) in seaweed-derived food products, within species between Ria de Aveiro Aquaculture (A) and wild (W) (Ria de Aveiro) of *Fucus* sp., *Gracilaria* sp. and *Ulva* sp.

Species	Origin	Total As (mg/kg DW)	Estimated iAs (mg/kg DW)	% of Regulatory Limit
*Fucus* sp.	A	31.9 ± 7.4	3.19 ± 0.74	106.30%
	W	26.3 ± 4.8	2.63 ± 0.48	87.70%
*Gracilaria* sp.	A	12.6 ± 6.48	1.26 ± 0.65	42.00%
	W	9.34 ± 4.40	0.93 ± 0.44	31.10%
*Ulva* sp.	A	1.61 ± 1.45	0.16 ± 0.15	5.40%
	W	1.30 ± 0.19	0.13 ± 0.02	4.30%

## Data Availability

All data supporting the findings of this study are available within the paper.

## Supplementary Materials

The following supporting information can be downloaded at: https://www.mdpi.com/article/10.3390/biology15110820/s1. The Supplementary Materials include three tables providing methodological and analytical details. Table S1: MRM transitions monitored for screening the lipophilic marine toxins. Oas—okadaic acid, AZAs—azaspiracid, YTX—yessotoxin, SPXs—spirolides, GYMs—gymnodimines and PnTXs—pinnatoxins in macroalgae samples from the Portuguese coast; Table S2: Elemental concentrations (mg/kg dry weight) obtained for certified reference materials TOR-3 and DORM-4 compared to their certified values. Limits of detection (LoD) and quantification (LoQ) are also presented for each element. Values are expressed as mean ± standard deviation.; Table S3: Elemental concentrations comparison between wild (W) and farmed (A) Ria de Aveiro samples (mg/kg Dw; mean ± STDEV) across macroalgae species. Copper (Cu), Bromine (Br), Strontium (Sr). In each column, different lowercase letters indicate statistically significant differences (Tukey HSD, *p* < 0.05).

## Abbreviations

The following abbreviations are used in this manuscript:

IPMA	Portuguese Institute for the Sea and Atmosphere
MARE	Marine and Environmental Sciences Centre
CIIMAR	Interdisciplinary Centre of Marine and Environmental Research
LA-REAL	Associated Laboratory
FCT-UNL	Faculty of Science and Technology, NOVA University Lisbon
SA	Sociedade Anónima
CCMAR	Centre of Marine Sciences
CIMAR LA	Centre of Marine Sciences Associated Laboratory
FAO	Food and Agriculture Organization
EFSA	European Food Safety Authority
CEVA	Regulatory Authority (seaweed standards context)
HABs	Harmful Algal Blooms
ASP	Amnesic Shellfish Poisoning
PSP	Paralytic Shellfish Poisoning
FW	Fresh Weight
DW	Dry Weight
XRF	X-ray Fluorescence
CRM	Certified Reference Material
LC-MSMS	Liquid Chromatography–Tandem Mass Spectrometry
HPLC-UV	High Performance Liquid Chromatography with UV detection
HPLC-FLD	High Performance Liquid Chromatography with Fluorescence Detection
DAD	Diode Array Detector
MRM	Multiple Reaction Monitoring
OAs	Okadaic Acids
AZAs	Azaspiracids
YTX	Yessotoxins
SPXs	Spirolides
GYMs	Gymnodimines
PnTXs	Pinnatoxins
SPX1	Spirolide toxin analogue
UL	Tolerable Upper Intake Level
PRI	Population Reference Intake
AI	Adequate Intake
ANOVA	Analysis of Variance
HSD	Honest Significant Difference
SD	Standard Deviation
REML	Restricted Maximum Likelihood
K	Potassium
Ca	Calcium
Mn	Manganese
Fe	Iron
Cu	Copper
Zn	Zinc
As	Arsenic
Br	Bromine
Sr	Strontium
C	Carbon
H	Hydrogen
O	Oxygen
Al	Aluminum
Ti	Titanium
SP	Spring
S	Summer
A	Autumn
W	Winter
