# Supplementary material for: Farmed and Wild Macroalgae as a Safe Source of Macro and Trace Elements
Source: Biology (Basel). 2026 May 22;15(11):820. doi: 10.3390/biology15110820 (PMC13255989; doi:10.3390/biology15110820)
Supplement: Supplementary file 1 [file biology-15-00820-s001.zip › biology-4269326-supplementary.pdf]

Farmed and wild macroalgae as a safe source of macro and trace elements

Tomás Chainho[1*], Rui Cereja[1,2], Alícia Pereira[3], Vera Marques[1], João C. Silva[4], Sofia Pessanha[4], Pedro Reis Costa[1,5], António Marques[1,3]

Supplementary material

Table S1- MRM transitions monitored for screening the lipophilic marine toxins. OAs – okadaic acid, AZAs - azaspiracid, YTX - yessotoxin, SPXs - spirolides, GYMs – gymnodimines and PnTXs – pinnatoxins in macroalgae samples from the Portuguese coast.

Group	Toxin	Precursor ion, m/z	Product ion, m/z	Ion polarity
OAs	OA	803.5	255.2	neg
		803.5	113.1	neg
	DTX1	817.5	255.2	neg
		817.5	113.1	neg
	DTX2	803.5	255.2	neg
		803.5	113.1	neg
YTXs	YTX	1141.6	458.3	neg
		1141.6	164.1	neg
	homo-YTX	1155.6	1075.6	neg
		1155.6	938.5	neg
	45-OH-YTX	1157.6	1077.6	neg
		1157.6	871.5	neg
	45-OH-homo-YTX	1171.6	1091.6	neg
		1171.6	869.5	neg
AZAs	AZA1	842.6	824.6	pos
		842.6	672.4	pos
	AZA2	856.5	838.5	pos
		856.5	672.4	pos
	AZA3	828.5	810.5	pos
		828.5	658.4	pos
PTX	PTX1	892.5	821.5	pos
		892.5	213.2	pos
	PTX2	876.5	823.5	pos
		876.5	213.2	pos
SPXs	13-desmethyl SPX-C	692.5	444.3	pos
		692.5	164.1	pos
	13,19-desmethylSPX-C	678.5	430.3	pos
		678.5	164.1	pos
GYMs	GYM-A	508.3	392.3	pos
		508.3	136.1	pos
PnTXs	PnTX A	712.4	458.3	pos
		712.4	164.1	pos
	PnTX BC	741.5	458.3	pos
		741.5	164.1	pos
PnTXs	PnTX D	782.5	446.3	pos
		782.5	164.1	pos
	PnTX E	784.5	446.3	pos
		784.5	164.1	pos
	PnTX F	766.5	446.3	pos
		766.5	164.1	pos
	PnTX G	694.5	458.1	pos
		694.5	164	pos
	PnTX A/B/C	831.1	458.1	pos
		831.1	164	pos

Table S2- Elemental concentrations (mg/kg dry weight) obtained for certified reference materials TOR-3 and DORM-4 compared to their certified values. Limits of detection (LoD) and quantification (LoQ) are also presented for each element. Values are expressed as mean ± standard deviation.

Elements	TOR-3		DORM-4		LoD	LoQ
	obtained	certifiedd	Obtained	certified		
K	-	-	10300 ± 700	15500 ± 1000	24	71
Ca	-	-	1700 ± 200	2360 ± 140	13	40
Mn	10 ± 1	15.6 ± 1.0	-	-	4	11
Fe	150 ± 15	179 ± 8	341± 20	343 ± 20	3	8
Cu	380 ± 40	497 ± 22	15 ± 2	15.7 ± 0.5	0.5	1.4
Zn	100 ± 10	136 ± 6	51 ± 6	51.6 ± 2.8	0.5	1.4
As	62± 7	59.5 ± 3.8	5.6 ± 0.8	6.90 ± 0.44	0.4	1.2
Sr	20 ± 3	36.5 ± 1.6	4.7 ± 0.6	10.1 ± 0.8	3	10

Table S3.- Elemental concentrations comparison between wild (W) and farmed (A) Ria de Aveiro samples (mg/kg Dw; mean ± STDEV) across macroalgae species. Copper (Cu), Bromine (Br), Strontium (Sr). In each column, different lowercase letters indicate statistically significant differences (Tukey HSD, $p < 0.05$).

Species	Origin	Cu	Br	Sr	Mn
***Fucus* sp.**	A	3.79 ± 1.43	358 ± 74.4	445 ± 67.1	263 ± 53.2
***Fucus* sp.**	W	3.10 ± 2.01	325 ± 55.9	423 ± 76.0	312 ± 130
***Gracilaria* sp.**	A	2.21 ± 0.950	281 ± 57.1	13.4 ± 2.00	304±195
***Gracilaria* sp.**	W	2.61 ± 0.870	277 ± 73.1	16.7 ± 8.04	245±310
***Ulva* sp.**	A	2.14 ± 1.09	176.61 ± 35.62	18.9 ± 5.79	90.1±119
***Ulva* sp.**	W	2.12 ± 1.24	167.28 ± 27.06	25.5 ± 9.15	54.9±44.8
